# μ-1,2-Di-4-pyridylethane-κ^2^ *N*:*N*′-bis­[bis­(*N*,*N*-diisopropyl­dithio­carbamato-κ^2^ *S*,*S*′)zinc(II)]

**DOI:** 10.1107/S1600536808010003

**Published:** 2008-04-18

**Authors:** Vanessa Avila, Edward R. T. Tiekink

**Affiliations:** a Department of Chemistry, The University of Texas at San Antonio, One UTSA Circle, San Antonio, Texas 78249-0698, USA

## Abstract

In the dinuclear title compound, [Zn{S_2_CN(*n*-Pr)_2_}_2_{(NC_5_H_4_)CH_2_CH_2_(C_5_H_4_N)}] or [Zn_2_(C_7_H_14_NS_2_)_4_(C_12_H_12_N_2_)], each Zn atom adopts a distorted trigonal–bipyramidal ZnNS_4_ geometry. The crystal structure involves intermolecular C—H⋯S hydrogen bonds.

## Related literature

For related structures, see: Lai *et al.* (2004 ▶); Chen *et al.* (2006 ▶); Benson *et al.* (2007 ▶). For related literature, see: Tiekink (2006 ▶). For structure analysis, see: Addison *et al.* (1984 ▶).
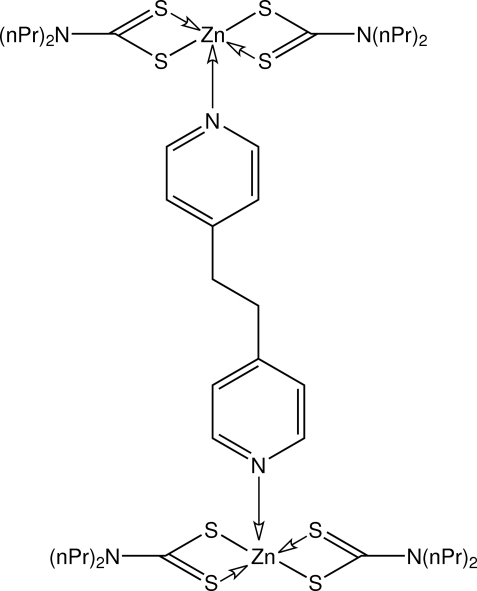


## Experimental

### 

#### Crystal data

[Zn_2_(C_7_H_14_NS_2_)_4_(C_12_H_12_N_2_)]
*M* *_r_* = 1020.22
Monoclinic, 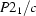

*a* = 18.645 (5) Å
*b* = 15.464 (5) Å
*c* = 17.567 (4) Å
β = 90.756 (11)°
*V* = 5064 (3) Å^3^
*Z* = 4
Mo *K*α radiation
μ = 1.31 mm^−1^
*T* = 98 (2) K
0.15 × 0.11 × 0.11 mm

#### Data collection

Rigaku AFC12K/SATURN724 diffractometer
Absorption correction: multi-scan (*ABSCOR*; Higashi, 1995 ▶) *T*_min_ = 0.757, *T*_max_ = 1 (expected range = 0.655–0.866)
52873 measured reflections
10501 independent reflections
9945 reflections with *I* > 2σ(*I*)
*R*_int_ = 0.040

#### Refinement

*R*[*F* ^2^ > 2σ(*F* ^2^)] = 0.040
*wR*(*F* ^2^) = 0.089
*S* = 1.14
10501 reflections
505 parameters
H-atom parameters constrained
Δρ_max_ = 0.47 e Å^−3^
Δρ_min_ = −0.41 e Å^−3^

### 

Data collection: *CrystalClear* (Rigaku, 2005 ▶); cell refinement: *CrystalClear*; data reduction: *CrystalClear*; program(s) used to solve structure: *PATTY* in *DIRDIF92* (Beurskens *et al.*, 1992 ▶); program(s) used to refine structure: *SHELXL97* (Sheldrick, 2008 ▶); molecular graphics: *ORTEPII* (Johnson, 1976 ▶) and *DIAMOND* (Brandenburg, 2006 ▶); software used to prepare material for publication: *SHELXL97*.

## Supplementary Material

Crystal structure: contains datablocks global, I. DOI: 10.1107/S1600536808010003/sj2481sup1.cif

Structure factors: contains datablocks I. DOI: 10.1107/S1600536808010003/sj2481Isup2.hkl

Additional supplementary materials:  crystallographic information; 3D view; checkCIF report

## Figures and Tables

**Table:** 

Zn1—S1	2.3627 (8)
Zn1—S2	2.5573 (8)
Zn1—S3	2.3437 (8)
Zn1—S4	2.5978 (8)
Zn1—N3	2.0882 (19)
Zn2—S5	2.3295 (8)
Zn2—S6	2.6166 (9)
Zn2—S7	2.3327 (9)
Zn2—S8	2.5829 (8)
Zn2—N4	2.0691 (19)

**Table:** 

N3—Zn1—S1	125.92 (6)
S2—Zn1—S4	164.37 (2)
S5—Zn2—S7	124.05 (3)
S6—Zn2—S8	167.27 (2)

**Table 2:** Hydrogen-bond geometry (Å, °)

*D*—H⋯*A*	*D*—H	H⋯*A*	*D*⋯*A*	*D*—H⋯*A*
C28—H28a⋯S1^i^	0.99	2.82	3.706 (3)	149
C40—H40b⋯S3^ii^	0.98	2.87	3.834 (3)	170

Symmetry codes: (i) 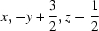; (ii) 
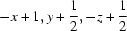
.

**Fig. 1.**
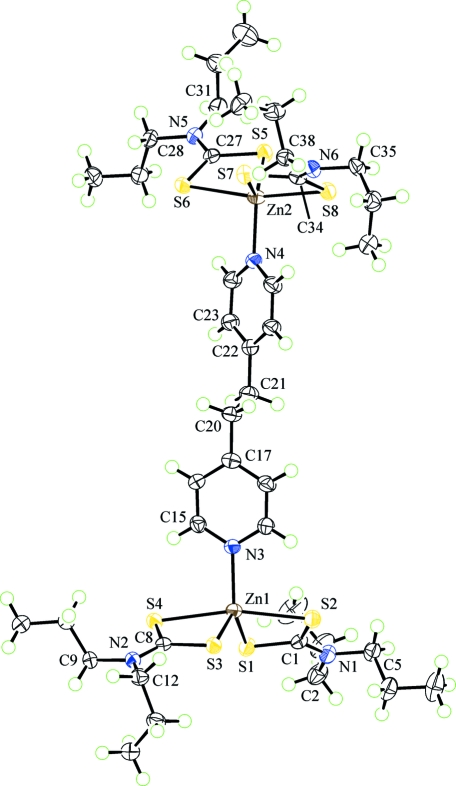
The molecular structure of (I) showing atom labelling scheme and displacement ellipsoids at the 50% probability level (arbitrary spheres for the H atoms). For clarity only the first atom of consecutively numbered iso-propyl groups and the N and two C atoms of the bispyridylethane ligand have been numbered.

**Fig. 2.**
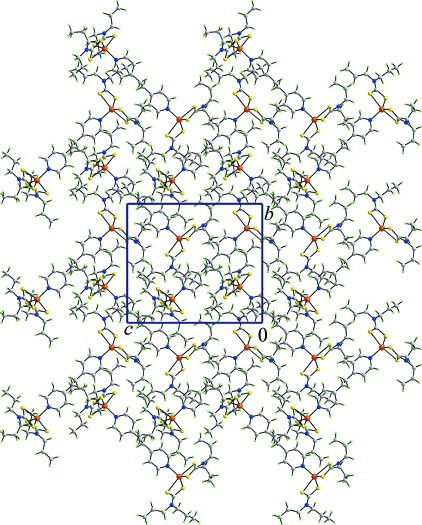
View of a layer in (I) highlighting the herringbone arrangement. Colour code: Zn (orange), S (yellow), O (red), N (blue), C (grey) & H (green).

**Fig. 3.**
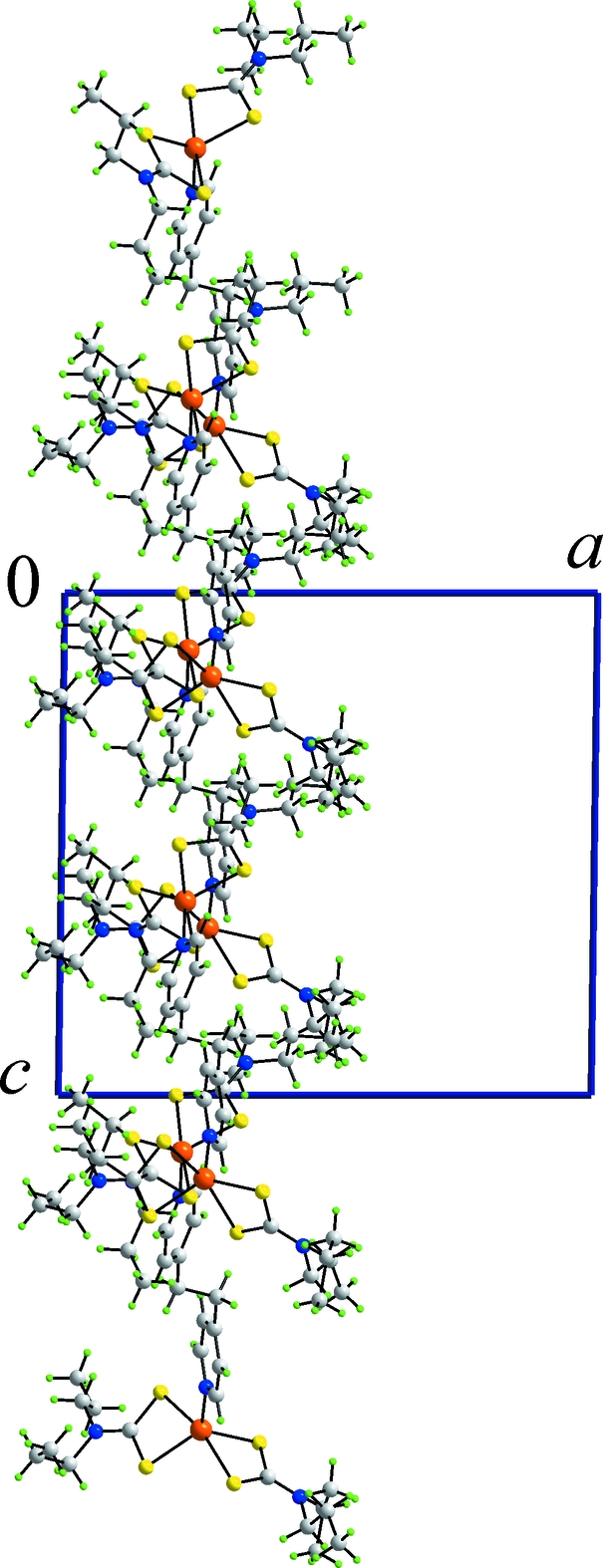
Side-on view of a layer in (I). Colour code as for Fig. 2.

## supplementary crystallographic information

### Comment

Interest in the title compound (I), Fig. 1 & Table 1, rests with an on-going investigation of the aggregation patterns of zinc and cadmium 1,1-dithiolates (Lai *et al.*, 2004, Chen *et al.*, 2006 & Benson *et al.*, 2007). The dimeric structure features two highly distorted trigonal bipyramidal NS_4_ coordination geometries with values of τ = 0.64 and 0.72 for Zn1 and Zn2, respectively (Addison *et al.*, 1984). The dithiocarbamate ligands coordinate forming almost symmetrical Zn—S bond distances in accord with expectation (Tiekink, 2006). The primary interactions between molecules are of the type C—H···S, Table 2. Each dimer participates in four such interactions. Dimers are arranged in an herringbone fashion in the *bc*-plane, Fig. 2. The resultant layers are basically flat with the n-propyl groups projecting above and below to inter-digitate with adjacent layers, Fig. 3.

### Experimental

The title compound was prepared by refluxing the parent zinc dithiocarbamate with 1,2-bis(4-pyridyl)ethane using a literature procedure (Lai *et al.*, 2004). Colourless crystals of (I) were isolated by the slow evaporation of an ethanol/methanol (1/1) solution; m.p. 451 - 453 K.

### Refinement

The H atoms were geometrically placed (C—H = 0.95–0.99 Å) and refined as riding with *U*_iso_(H) = 1.2*U*_eq_(C) or 1.5*U*_eq_(methyl C).

### Crystal data

**Table:**

[Zn_2_(C_7_H_14_NS_2_)_4_(C_12_H_12_N_2_)]	*F*(000) = 2152
*M**_r_* = 1020.22	*D*_x_ = 1.338 Mg m^−^^3^
Monoclinic, *P*2_1_/*c*	Mo *K*α radiation, λ = 0.71073 Å
Hall symbol: -P 2ybc	Cell parameters from 17064 reflections
*a* = 18.645 (5) Å	θ = 2.1–30.5°
*b* = 15.464 (5) Å	µ = 1.31 mm^−^^1^
*c* = 17.567 (4) Å	*T* = 98 K
β = 90.756 (11)°	Block, colourless
*V* = 5064 (3) Å^3^	0.15 × 0.11 × 0.11 mm
*Z* = 4	

### Data collection

**Table:**

Rigaku AFC12K/SATURN724 diffractometer	10501 independent reflections
Radiation source: fine-focus sealed tube	9945 reflections with *I* > 2σ(*I*)
graphite	*R*_int_ = 0.040
ω scans	θ_max_ = 26.5°, θ_min_ = 1.8°
Absorption correction: multi-scan (*ABSCOR*; Higashi, 1995)	*h* = −20→23
*T*_min_ = 0.757, *T*_max_ = 1	*k* = −19→19
52873 measured reflections	*l* = −22→22

### Refinement

**Table:**

Refinement on *F*^2^	Primary atom site location: structure-invariant direct methods
Least-squares matrix: full	Secondary atom site location: difference Fourier map
*R*[*F*^2^ > 2σ(*F*^2^)] = 0.040	Hydrogen site location: inferred from neighbouring sites
*wR*(*F*^2^) = 0.089	H-atom parameters constrained
*S* = 1.14	*w* = 1/[σ^2^(*F*_o_^2^) + (0.0347*P*)^2^ + 4.0808*P*] where *P* = (*F*_o_^2^ + 2*F*_c_^2^)/3
10501 reflections	(Δ/σ)_max_ = 0.001
505 parameters	Δρ_max_ = 0.47 e Å^−^^3^
0 restraints	Δρ_min_ = −0.41 e Å^−^^3^

### Special details

**Table:**

Geometry. All e.s.d.'s (except the e.s.d. in the dihedral angle between two l.s. planes) are estimated using the full covariance matrix. The cell e.s.d.'s are taken into account individually in the estimation of e.s.d.'s in distances, angles and torsion angles; correlations between e.s.d.'s in cell parameters are only used when they are defined by crystal symmetry. An approximate (isotropic) treatment of cell e.s.d.'s is used for estimating e.s.d.'s involving l.s. planes.
Refinement. Refinement of *F*^2^ against ALL reflections. The weighted *R*-factor *wR* and goodness of fit *S* are based on *F*^2^, conventional *R*-factors *R* are based on *F*, with *F* set to zero for negative *F*^2^. The threshold expression of *F*^2^ > σ(*F*^2^) is used only for calculating *R*-factors(gt) *etc*. and is not relevant to the choice of reflections for refinement. *R*-factors based on *F*^2^ are statistically about twice as large as those based on *F*, and *R*- factors based on ALL data will be even larger.

### Fractional atomic coordinates and isotropic or equivalent isotropic displacement parameters (Å^2^)

**Table:**

	*x*	*y*	*z*	*U*_iso_*/*U*_eq_	
Zn1	0.276298 (14)	0.197149 (17)	0.666864 (15)	0.01860 (7)	
Zn2	0.233401 (14)	0.792692 (17)	0.113503 (15)	0.01919 (7)	
S1	0.17394 (3)	0.17012 (4)	0.74108 (3)	0.02267 (13)	
S2	0.19934 (3)	0.08862 (4)	0.59234 (3)	0.02227 (13)	
S3	0.38566 (3)	0.12770 (4)	0.69274 (3)	0.02016 (12)	
S4	0.33916 (3)	0.28608 (4)	0.77400 (3)	0.01942 (12)	
S5	0.14013 (3)	0.88860 (4)	0.08654 (3)	0.02158 (13)	
S6	0.25034 (3)	0.92616 (4)	0.20351 (3)	0.02157 (13)	
S7	0.34319 (3)	0.79323 (4)	0.05134 (3)	0.02127 (13)	
S8	0.22111 (3)	0.68586 (4)	0.00107 (3)	0.02082 (13)	
N1	0.07846 (11)	0.06823 (13)	0.67052 (11)	0.0228 (4)	
N2	0.46332 (10)	0.20599 (12)	0.80062 (11)	0.0192 (4)	
N3	0.28479 (10)	0.29088 (12)	0.58188 (11)	0.0182 (4)	
N4	0.23109 (10)	0.70372 (12)	0.20140 (11)	0.0185 (4)	
N5	0.14157 (10)	1.03193 (12)	0.17162 (11)	0.0203 (4)	
N6	0.35081 (10)	0.68252 (12)	−0.06437 (11)	0.0202 (4)	
C1	0.14307 (12)	0.10457 (14)	0.66783 (13)	0.0197 (5)	
C2	0.02924 (14)	0.07935 (17)	0.73493 (15)	0.0290 (6)	
H2A	0.0570	0.1002	0.7797	0.035*	
H2B	0.0086	0.0224	0.7481	0.035*	
C3	−0.03145 (15)	0.14235 (18)	0.71824 (18)	0.0370 (7)	
H3A	−0.0579	0.1227	0.6722	0.044*	
H3B	−0.0652	0.1414	0.7612	0.044*	
C4	−0.00637 (16)	0.23467 (19)	0.7060 (2)	0.0501 (9)	
H4A	−0.0478	0.2715	0.6940	0.075*	
H4B	0.0273	0.2363	0.6636	0.075*	
H4C	0.0176	0.2558	0.7523	0.075*	
C5	0.05257 (13)	0.00974 (15)	0.60992 (14)	0.0247 (5)	
H5A	0.0719	0.0292	0.5606	0.030*	
H5B	−0.0004	0.0132	0.6066	0.030*	
C6	0.07454 (14)	−0.08379 (16)	0.62354 (16)	0.0308 (6)	
H6A	0.0580	−0.1022	0.6744	0.037*	
H6B	0.1275	−0.0881	0.6232	0.037*	
C7	0.04309 (15)	−0.14398 (19)	0.56328 (19)	0.0418 (7)	
H7A	0.0579	−0.2035	0.5742	0.063*	
H7B	0.0605	−0.1270	0.5130	0.063*	
H7C	−0.0094	−0.1402	0.5637	0.063*	
C8	0.40247 (12)	0.20721 (14)	0.76077 (13)	0.0186 (5)	
C9	0.47860 (13)	0.27132 (15)	0.85971 (13)	0.0209 (5)	
H9A	0.5103	0.2455	0.8992	0.025*	
H9B	0.4331	0.2878	0.8843	0.025*	
C10	0.51413 (15)	0.35234 (16)	0.82855 (14)	0.0281 (6)	
H10A	0.5567	0.3357	0.7988	0.034*	
H10B	0.4801	0.3825	0.7939	0.034*	
C11	0.53706 (15)	0.41336 (16)	0.89228 (15)	0.0302 (6)	
H11A	0.5593	0.4650	0.8704	0.045*	
H11B	0.5717	0.3841	0.9259	0.045*	
H11C	0.4950	0.4304	0.9215	0.045*	
C12	0.52046 (12)	0.14240 (15)	0.78581 (13)	0.0213 (5)	
H12A	0.5668	0.1663	0.8043	0.026*	
H12B	0.5241	0.1338	0.7301	0.026*	
C13	0.50823 (13)	0.05467 (15)	0.82364 (13)	0.0237 (5)	
H13A	0.4664	0.0264	0.7988	0.028*	
H13B	0.5507	0.0177	0.8149	0.028*	
C14	0.49519 (14)	0.05979 (16)	0.90901 (14)	0.0270 (5)	
H14A	0.4868	0.0016	0.9290	0.041*	
H14B	0.4531	0.0960	0.9184	0.041*	
H14C	0.5373	0.0851	0.9345	0.041*	
C15	0.30356 (12)	0.37329 (15)	0.59697 (13)	0.0204 (5)	
H15	0.3127	0.3895	0.6484	0.025*	
C16	0.31004 (12)	0.43544 (15)	0.54064 (13)	0.0207 (5)	
H16	0.3226	0.4931	0.5539	0.025*	
C17	0.29832 (12)	0.41359 (15)	0.46541 (13)	0.0215 (5)	
C18	0.27989 (14)	0.32803 (16)	0.44944 (14)	0.0246 (5)	
H18	0.2717	0.3101	0.3983	0.029*	
C19	0.27354 (13)	0.26952 (15)	0.50843 (13)	0.0231 (5)	
H19	0.2606	0.2116	0.4966	0.028*	
C20	0.30233 (13)	0.47978 (16)	0.40236 (14)	0.0251 (5)	
H20A	0.3227	0.4526	0.3564	0.030*	
H20B	0.3346	0.5275	0.4183	0.030*	
C21	0.22824 (13)	0.51607 (15)	0.38322 (13)	0.0232 (5)	
H21A	0.1961	0.4680	0.3679	0.028*	
H21B	0.2082	0.5432	0.4293	0.028*	
C22	0.22989 (12)	0.58203 (15)	0.32004 (13)	0.0205 (5)	
C23	0.20409 (13)	0.66545 (15)	0.33017 (13)	0.0225 (5)	
H23	0.1854	0.6825	0.3780	0.027*	
C24	0.20565 (13)	0.72358 (15)	0.27055 (13)	0.0224 (5)	
H24	0.1879	0.7804	0.2788	0.027*	
C25	0.25688 (13)	0.62323 (15)	0.19170 (13)	0.0227 (5)	
H25	0.2756	0.6081	0.1435	0.027*	
C26	0.25737 (13)	0.56184 (15)	0.24854 (13)	0.0224 (5)	
H26	0.2763	0.5059	0.2392	0.027*	
C27	0.17453 (12)	0.95762 (14)	0.15605 (13)	0.0193 (5)	
C28	0.16746 (13)	1.09056 (15)	0.23204 (13)	0.0211 (5)	
H28A	0.1553	1.1508	0.2178	0.025*	
H28B	0.2204	1.0863	0.2360	0.025*	
C29	0.13528 (13)	1.07032 (16)	0.30966 (14)	0.0251 (5)	
H29A	0.0824	1.0757	0.3064	0.030*	
H29B	0.1470	1.0100	0.3241	0.030*	
C30	0.16433 (14)	1.13174 (17)	0.37031 (14)	0.0285 (6)	
H30A	0.1434	1.1173	0.4196	0.043*	
H30B	0.1517	1.1913	0.3567	0.043*	
H30C	0.2166	1.1262	0.3738	0.043*	
C31	0.08007 (13)	1.06331 (15)	0.12656 (14)	0.0242 (5)	
H31A	0.0485	1.0981	0.1595	0.029*	
H31B	0.0521	1.0133	0.1073	0.029*	
C32	0.10403 (14)	1.11850 (17)	0.05937 (15)	0.0304 (6)	
H32A	0.1276	1.1716	0.0788	0.036*	
H32B	0.1397	1.0859	0.0295	0.036*	
C33	0.04147 (17)	1.1435 (2)	0.00780 (19)	0.0479 (8)	
H33A	0.0589	1.1792	−0.0342	0.072*	
H33B	0.0063	1.1763	0.0370	0.072*	
H33C	0.0188	1.0911	−0.0128	0.072*	
C34	0.30891 (12)	0.71559 (15)	−0.01075 (13)	0.0191 (5)	
C35	0.32439 (13)	0.62269 (16)	−0.12376 (13)	0.0235 (5)	
H35A	0.3371	0.6462	−0.1742	0.028*	
H35B	0.2714	0.6199	−0.1214	0.028*	
C36	0.35456 (14)	0.53096 (16)	−0.11662 (15)	0.0283 (5)	
H36A	0.3379	0.4965	−0.1609	0.034*	
H36B	0.4076	0.5337	−0.1183	0.034*	
C37	0.33268 (16)	0.48523 (17)	−0.04401 (15)	0.0340 (6)	
H37A	0.3537	0.4271	−0.0428	0.051*	
H37B	0.2803	0.4808	−0.0426	0.051*	
H37C	0.3500	0.5183	0.0002	0.051*	
C38	0.42600 (13)	0.71040 (15)	−0.07166 (14)	0.0209 (5)	
H38A	0.4537	0.6631	−0.0952	0.025*	
H38B	0.4464	0.7206	−0.0201	0.025*	
C39	0.43520 (14)	0.79207 (16)	−0.11890 (15)	0.0269 (5)	
H39A	0.4223	0.7801	−0.1727	0.032*	
H39B	0.4029	0.8379	−0.1000	0.032*	
C40	0.51250 (14)	0.82263 (17)	−0.11344 (16)	0.0310 (6)	
H40A	0.5183	0.8750	−0.1442	0.046*	
H40B	0.5443	0.7772	−0.1324	0.046*	
H40C	0.5248	0.8354	−0.0602	0.046*	

### Atomic displacement parameters (Å^2^)

**Table:**

	*U*^11^	*U*^22^	*U*^33^	*U*^12^	*U*^13^	*U*^23^
Zn1	0.01984 (14)	0.01810 (14)	0.01781 (14)	−0.00116 (10)	−0.00162 (10)	0.00323 (10)
Zn2	0.02074 (14)	0.01852 (14)	0.01832 (14)	0.00293 (10)	0.00090 (11)	0.00377 (10)
S1	0.0264 (3)	0.0201 (3)	0.0216 (3)	−0.0050 (2)	0.0031 (2)	−0.0030 (2)
S2	0.0238 (3)	0.0234 (3)	0.0196 (3)	−0.0045 (2)	0.0012 (2)	−0.0015 (2)
S3	0.0239 (3)	0.0168 (3)	0.0196 (3)	0.0009 (2)	−0.0034 (2)	−0.0014 (2)
S4	0.0219 (3)	0.0182 (3)	0.0180 (3)	0.0021 (2)	−0.0028 (2)	−0.0004 (2)
S5	0.0230 (3)	0.0191 (3)	0.0226 (3)	0.0028 (2)	−0.0059 (2)	−0.0028 (2)
S6	0.0253 (3)	0.0186 (3)	0.0206 (3)	0.0018 (2)	−0.0063 (2)	0.0012 (2)
S7	0.0196 (3)	0.0236 (3)	0.0207 (3)	−0.0013 (2)	0.0001 (2)	−0.0038 (2)
S8	0.0199 (3)	0.0237 (3)	0.0188 (3)	−0.0016 (2)	−0.0010 (2)	−0.0006 (2)
N1	0.0235 (10)	0.0208 (10)	0.0240 (10)	−0.0029 (8)	0.0007 (8)	−0.0021 (8)
N2	0.0209 (10)	0.0181 (10)	0.0186 (10)	0.0005 (7)	−0.0034 (8)	−0.0003 (8)
N3	0.0215 (10)	0.0157 (9)	0.0173 (10)	0.0007 (7)	−0.0005 (8)	0.0018 (7)
N4	0.0217 (10)	0.0155 (9)	0.0183 (10)	0.0011 (7)	−0.0003 (8)	0.0000 (7)
N5	0.0232 (10)	0.0170 (9)	0.0207 (10)	−0.0009 (8)	−0.0009 (8)	−0.0008 (8)
N6	0.0211 (10)	0.0218 (10)	0.0177 (10)	0.0006 (8)	−0.0010 (8)	−0.0017 (8)
C1	0.0232 (11)	0.0142 (11)	0.0218 (11)	−0.0016 (9)	−0.0009 (9)	0.0027 (9)
C2	0.0299 (13)	0.0257 (13)	0.0317 (14)	−0.0072 (11)	0.0108 (11)	−0.0016 (11)
C3	0.0294 (14)	0.0285 (14)	0.0534 (18)	−0.0032 (11)	0.0134 (13)	−0.0024 (13)
C4	0.0307 (15)	0.0302 (16)	0.090 (3)	0.0008 (12)	0.0138 (16)	0.0025 (16)
C5	0.0230 (12)	0.0219 (12)	0.0291 (13)	−0.0040 (10)	−0.0019 (10)	−0.0020 (10)
C6	0.0277 (13)	0.0235 (13)	0.0410 (15)	−0.0013 (10)	−0.0029 (12)	−0.0059 (11)
C7	0.0319 (15)	0.0314 (15)	0.062 (2)	−0.0014 (12)	−0.0045 (14)	−0.0186 (14)
C8	0.0231 (12)	0.0171 (11)	0.0155 (11)	−0.0044 (9)	−0.0003 (9)	0.0040 (9)
C9	0.0238 (12)	0.0196 (11)	0.0191 (11)	−0.0026 (9)	−0.0054 (9)	−0.0006 (9)
C10	0.0375 (14)	0.0230 (13)	0.0237 (13)	−0.0076 (11)	−0.0072 (11)	0.0033 (10)
C11	0.0390 (15)	0.0215 (13)	0.0300 (14)	−0.0059 (11)	−0.0076 (11)	0.0035 (11)
C12	0.0201 (11)	0.0210 (12)	0.0229 (12)	0.0026 (9)	−0.0007 (9)	0.0017 (9)
C13	0.0278 (12)	0.0219 (12)	0.0215 (12)	0.0037 (10)	−0.0017 (10)	0.0018 (10)
C14	0.0329 (14)	0.0243 (13)	0.0238 (13)	−0.0027 (10)	−0.0020 (10)	0.0027 (10)
C15	0.0238 (12)	0.0207 (12)	0.0168 (11)	0.0008 (9)	−0.0003 (9)	−0.0019 (9)
C16	0.0252 (12)	0.0160 (11)	0.0209 (11)	−0.0022 (9)	−0.0009 (9)	0.0020 (9)
C17	0.0215 (11)	0.0230 (12)	0.0201 (11)	0.0025 (9)	0.0003 (9)	0.0047 (9)
C18	0.0348 (14)	0.0227 (12)	0.0162 (11)	0.0011 (10)	0.0006 (10)	0.0008 (10)
C19	0.0326 (13)	0.0179 (11)	0.0188 (11)	0.0005 (10)	−0.0015 (10)	−0.0018 (9)
C20	0.0293 (13)	0.0259 (13)	0.0201 (12)	−0.0016 (10)	−0.0009 (10)	0.0073 (10)
C21	0.0308 (13)	0.0210 (12)	0.0178 (11)	0.0025 (10)	0.0014 (10)	0.0027 (9)
C22	0.0245 (12)	0.0183 (11)	0.0187 (11)	−0.0005 (9)	−0.0004 (9)	0.0009 (9)
C23	0.0300 (13)	0.0196 (12)	0.0180 (11)	0.0010 (10)	0.0021 (10)	−0.0008 (9)
C24	0.0275 (12)	0.0173 (11)	0.0224 (12)	0.0034 (9)	0.0024 (10)	−0.0011 (9)
C25	0.0279 (12)	0.0222 (12)	0.0180 (11)	0.0039 (10)	−0.0003 (10)	−0.0002 (9)
C26	0.0297 (13)	0.0173 (11)	0.0201 (12)	0.0052 (9)	−0.0003 (10)	−0.0001 (9)
C27	0.0234 (11)	0.0164 (11)	0.0182 (11)	−0.0028 (9)	0.0012 (9)	0.0026 (9)
C28	0.0236 (12)	0.0180 (11)	0.0218 (12)	−0.0036 (9)	0.0017 (9)	−0.0030 (9)
C29	0.0266 (12)	0.0241 (12)	0.0247 (13)	−0.0038 (10)	0.0009 (10)	−0.0021 (10)
C30	0.0329 (14)	0.0316 (14)	0.0209 (12)	−0.0039 (11)	−0.0009 (10)	−0.0016 (11)
C31	0.0203 (11)	0.0198 (12)	0.0326 (13)	0.0035 (9)	−0.0046 (10)	−0.0018 (10)
C32	0.0289 (13)	0.0297 (14)	0.0324 (14)	0.0030 (11)	−0.0068 (11)	0.0060 (11)
C33	0.0469 (18)	0.0470 (19)	0.0491 (19)	0.0063 (15)	−0.0201 (15)	0.0101 (15)
C34	0.0211 (11)	0.0200 (11)	0.0163 (11)	0.0024 (9)	−0.0018 (9)	0.0041 (9)
C35	0.0274 (12)	0.0273 (13)	0.0157 (11)	0.0022 (10)	−0.0020 (9)	−0.0038 (10)
C36	0.0313 (13)	0.0240 (13)	0.0295 (13)	0.0010 (10)	−0.0016 (11)	−0.0045 (11)
C37	0.0438 (16)	0.0254 (13)	0.0326 (15)	−0.0059 (12)	−0.0059 (12)	0.0007 (11)
C38	0.0213 (12)	0.0215 (12)	0.0200 (12)	0.0003 (9)	0.0011 (9)	0.0001 (9)
C39	0.0314 (14)	0.0252 (13)	0.0243 (13)	0.0017 (10)	0.0077 (11)	0.0031 (10)
C40	0.0348 (14)	0.0249 (13)	0.0336 (15)	−0.0038 (11)	0.0114 (12)	−0.0045 (11)

### Geometric parameters (Å, °)

**Table:**

Zn1—S1	2.3627 (8)	C13—H13A	0.9900
Zn1—S2	2.5573 (8)	C13—H13B	0.9900
Zn1—S3	2.3437 (8)	C14—H14A	0.9800
Zn1—S4	2.5978 (8)	C14—H14B	0.9800
Zn1—N3	2.0882 (19)	C14—H14C	0.9800
Zn2—S5	2.3295 (8)	C15—C16	1.386 (3)
Zn2—S6	2.6166 (9)	C15—H15	0.9500
Zn2—S7	2.3327 (9)	C16—C17	1.379 (3)
Zn2—S8	2.5829 (8)	C16—H16	0.9500
Zn2—N4	2.0691 (19)	C17—C18	1.394 (3)
S1—C1	1.731 (2)	C17—C20	1.511 (3)
S2—C1	1.720 (2)	C18—C19	1.382 (3)
S3—C8	1.740 (2)	C18—H18	0.9500
S4—C8	1.715 (2)	C19—H19	0.9500
S5—C27	1.738 (2)	C20—C21	1.524 (3)
S6—C27	1.702 (2)	C20—H20A	0.9900
S7—C34	1.738 (2)	C20—H20B	0.9900
S8—C34	1.716 (2)	C21—C22	1.508 (3)
N1—C1	1.331 (3)	C21—H21A	0.9900
N1—C5	1.473 (3)	C21—H21B	0.9900
N1—C2	1.476 (3)	C22—C23	1.389 (3)
N2—C8	1.326 (3)	C22—C26	1.398 (3)
N2—C9	1.474 (3)	C23—C24	1.381 (3)
N2—C12	1.475 (3)	C23—H23	0.9500
N3—C19	1.346 (3)	C24—H24	0.9500
N3—C15	1.347 (3)	C25—C26	1.378 (3)
N4—C24	1.345 (3)	C25—H25	0.9500
N4—C25	1.346 (3)	C26—H26	0.9500
N5—C27	1.333 (3)	C28—C29	1.529 (3)
N5—C31	1.467 (3)	C28—H28A	0.9900
N5—C28	1.473 (3)	C28—H28B	0.9900
N6—C34	1.334 (3)	C29—C30	1.522 (3)
N6—C38	1.474 (3)	C29—H29A	0.9900
N6—C35	1.474 (3)	C29—H29B	0.9900
C2—C3	1.519 (4)	C30—H30A	0.9800
C2—H2A	0.9900	C30—H30B	0.9800
C2—H2B	0.9900	C30—H30C	0.9800
C3—C4	1.519 (4)	C31—C32	1.528 (4)
C3—H3A	0.9900	C31—H31A	0.9900
C3—H3B	0.9900	C31—H31B	0.9900
C4—H4A	0.9800	C32—C33	1.517 (4)
C4—H4B	0.9800	C32—H32A	0.9900
C4—H4C	0.9800	C32—H32B	0.9900
C5—C6	1.521 (3)	C33—H33A	0.9800
C5—H5A	0.9900	C33—H33B	0.9800
C5—H5B	0.9900	C33—H33C	0.9800
C6—C7	1.521 (4)	C35—C36	1.530 (3)
C6—H6A	0.9900	C35—H35A	0.9900
C6—H6B	0.9900	C35—H35B	0.9900
C7—H7A	0.9800	C36—C37	1.519 (4)
C7—H7B	0.9800	C36—H36A	0.9900
C7—H7C	0.9800	C36—H36B	0.9900
C9—C10	1.522 (3)	C37—H37A	0.9800
C9—H9A	0.9900	C37—H37B	0.9800
C9—H9B	0.9900	C37—H37C	0.9800
C10—C11	1.521 (3)	C38—C39	1.522 (3)
C10—H10A	0.9900	C38—H38A	0.9900
C10—H10B	0.9900	C38—H38B	0.9900
C11—H11A	0.9800	C39—C40	1.519 (4)
C11—H11B	0.9800	C39—H39A	0.9900
C11—H11C	0.9800	C39—H39B	0.9900
C12—C13	1.529 (3)	C40—H40A	0.9800
C12—H12A	0.9900	C40—H40B	0.9800
C12—H12B	0.9900	C40—H40C	0.9800
C13—C14	1.524 (3)		
			
N3—Zn1—S3	112.50 (6)	H14A—C14—H14C	109.5
N3—Zn1—S1	125.92 (6)	H14B—C14—H14C	109.5
S3—Zn1—S1	121.26 (3)	N3—C15—C16	122.7 (2)
N3—Zn1—S2	97.87 (6)	N3—C15—H15	118.6
S3—Zn1—S2	106.22 (3)	C16—C15—H15	118.6
S1—Zn1—S2	73.42 (3)	C17—C16—C15	120.0 (2)
N3—Zn1—S4	96.47 (6)	C17—C16—H16	120.0
S3—Zn1—S4	73.68 (3)	C15—C16—H16	120.0
S1—Zn1—S4	93.09 (3)	C16—C17—C18	117.4 (2)
S2—Zn1—S4	164.37 (2)	C16—C17—C20	121.9 (2)
N4—Zn2—S5	123.52 (6)	C18—C17—C20	120.6 (2)
N4—Zn2—S7	112.26 (6)	C19—C18—C17	119.6 (2)
S5—Zn2—S7	124.05 (3)	C19—C18—H18	120.2
N4—Zn2—S8	98.21 (6)	C17—C18—H18	120.2
S5—Zn2—S8	101.15 (3)	N3—C19—C18	123.0 (2)
S7—Zn2—S8	73.37 (2)	N3—C19—H19	118.5
N4—Zn2—S6	94.42 (6)	C18—C19—H19	118.5
S5—Zn2—S6	72.81 (3)	C17—C20—C21	111.0 (2)
S7—Zn2—S6	100.40 (2)	C17—C20—H20A	109.4
S6—Zn2—S8	167.27 (2)	C21—C20—H20A	109.4
C1—S1—Zn1	87.50 (8)	C17—C20—H20B	109.4
C1—S2—Zn1	81.66 (8)	C21—C20—H20B	109.4
C8—S3—Zn1	87.58 (8)	H20A—C20—H20B	108.0
C8—S4—Zn1	80.22 (8)	C22—C21—C20	112.6 (2)
C27—S5—Zn2	88.89 (8)	C22—C21—H21A	109.1
C27—S6—Zn2	80.60 (8)	C20—C21—H21A	109.1
C34—S7—Zn2	88.49 (8)	C22—C21—H21B	109.1
C34—S8—Zn2	81.14 (8)	C20—C21—H21B	109.1
C1—N1—C5	121.4 (2)	H21A—C21—H21B	107.8
C1—N1—C2	123.4 (2)	C23—C22—C26	117.0 (2)
C5—N1—C2	115.10 (19)	C23—C22—C21	121.6 (2)
C8—N2—C9	121.23 (19)	C26—C22—C21	121.4 (2)
C8—N2—C12	122.10 (19)	C24—C23—C22	119.8 (2)
C9—N2—C12	116.61 (18)	C24—C23—H23	120.1
C19—N3—C15	117.24 (19)	C22—C23—H23	120.1
C19—N3—Zn1	120.18 (15)	N4—C24—C23	123.2 (2)
C15—N3—Zn1	122.54 (15)	N4—C24—H24	118.4
C24—N4—C25	117.1 (2)	C23—C24—H24	118.4
C24—N4—Zn2	122.21 (15)	N4—C25—C26	123.0 (2)
C25—N4—Zn2	120.62 (15)	N4—C25—H25	118.5
C27—N5—C31	122.23 (19)	C26—C25—H25	118.5
C27—N5—C28	122.1 (2)	C25—C26—C22	119.9 (2)
C31—N5—C28	115.58 (19)	C25—C26—H26	120.1
C34—N6—C38	121.05 (19)	C22—C26—H26	120.1
C34—N6—C35	123.2 (2)	N5—C27—S6	121.86 (17)
C38—N6—C35	115.55 (19)	N5—C27—S5	120.44 (18)
N1—C1—S2	121.91 (18)	S6—C27—S5	117.70 (13)
N1—C1—S1	120.88 (18)	N5—C28—C29	112.82 (19)
S2—C1—S1	117.21 (13)	N5—C28—H28A	109.0
N1—C2—C3	113.3 (2)	C29—C28—H28A	109.0
N1—C2—H2A	108.9	N5—C28—H28B	109.0
C3—C2—H2A	108.9	C29—C28—H28B	109.0
N1—C2—H2B	108.9	H28A—C28—H28B	107.8
C3—C2—H2B	108.9	C30—C29—C28	110.9 (2)
H2A—C2—H2B	107.7	C30—C29—H29A	109.5
C4—C3—C2	113.6 (2)	C28—C29—H29A	109.5
C4—C3—H3A	108.8	C30—C29—H29B	109.5
C2—C3—H3A	108.8	C28—C29—H29B	109.5
C4—C3—H3B	108.8	H29A—C29—H29B	108.1
C2—C3—H3B	108.8	C29—C30—H30A	109.5
H3A—C3—H3B	107.7	C29—C30—H30B	109.5
C3—C4—H4A	109.5	H30A—C30—H30B	109.5
C3—C4—H4B	109.5	C29—C30—H30C	109.5
H4A—C4—H4B	109.5	H30A—C30—H30C	109.5
C3—C4—H4C	109.5	H30B—C30—H30C	109.5
H4A—C4—H4C	109.5	N5—C31—C32	111.5 (2)
H4B—C4—H4C	109.5	N5—C31—H31A	109.3
N1—C5—C6	112.6 (2)	C32—C31—H31A	109.3
N1—C5—H5A	109.1	N5—C31—H31B	109.3
C6—C5—H5A	109.1	C32—C31—H31B	109.3
N1—C5—H5B	109.1	H31A—C31—H31B	108.0
C6—C5—H5B	109.1	C33—C32—C31	111.9 (2)
H5A—C5—H5B	107.8	C33—C32—H32A	109.2
C5—C6—C7	111.8 (2)	C31—C32—H32A	109.2
C5—C6—H6A	109.3	C33—C32—H32B	109.2
C7—C6—H6A	109.3	C31—C32—H32B	109.2
C5—C6—H6B	109.3	H32A—C32—H32B	107.9
C7—C6—H6B	109.3	C32—C33—H33A	109.5
H6A—C6—H6B	107.9	C32—C33—H33B	109.5
C6—C7—H7A	109.5	H33A—C33—H33B	109.5
C6—C7—H7B	109.5	C32—C33—H33C	109.5
H7A—C7—H7B	109.5	H33A—C33—H33C	109.5
C6—C7—H7C	109.5	H33B—C33—H33C	109.5
H7A—C7—H7C	109.5	N6—C34—S8	123.47 (18)
H7B—C7—H7C	109.5	N6—C34—S7	119.62 (18)
N2—C8—S4	121.63 (17)	S8—C34—S7	116.92 (13)
N2—C8—S3	119.85 (18)	N6—C35—C36	113.9 (2)
S4—C8—S3	118.52 (13)	N6—C35—H35A	108.8
N2—C9—C10	113.1 (2)	C36—C35—H35A	108.8
N2—C9—H9A	109.0	N6—C35—H35B	108.8
C10—C9—H9A	109.0	C36—C35—H35B	108.8
N2—C9—H9B	109.0	H35A—C35—H35B	107.7
C10—C9—H9B	109.0	C37—C36—C35	113.4 (2)
H9A—C9—H9B	107.8	C37—C36—H36A	108.9
C11—C10—C9	111.4 (2)	C35—C36—H36A	108.9
C11—C10—H10A	109.3	C37—C36—H36B	108.9
C9—C10—H10A	109.3	C35—C36—H36B	108.9
C11—C10—H10B	109.3	H36A—C36—H36B	107.7
C9—C10—H10B	109.3	C36—C37—H37A	109.5
H10A—C10—H10B	108.0	C36—C37—H37B	109.5
C10—C11—H11A	109.5	H37A—C37—H37B	109.5
C10—C11—H11B	109.5	C36—C37—H37C	109.5
H11A—C11—H11B	109.5	H37A—C37—H37C	109.5
C10—C11—H11C	109.5	H37B—C37—H37C	109.5
H11A—C11—H11C	109.5	N6—C38—C39	113.84 (19)
H11B—C11—H11C	109.5	N6—C38—H38A	108.8
N2—C12—C13	113.74 (19)	C39—C38—H38A	108.8
N2—C12—H12A	108.8	N6—C38—H38B	108.8
C13—C12—H12A	108.8	C39—C38—H38B	108.8
N2—C12—H12B	108.8	H38A—C38—H38B	107.7
C13—C12—H12B	108.8	C40—C39—C38	109.7 (2)
H12A—C12—H12B	107.7	C40—C39—H39A	109.7
C14—C13—C12	114.1 (2)	C38—C39—H39A	109.7
C14—C13—H13A	108.7	C40—C39—H39B	109.7
C12—C13—H13A	108.7	C38—C39—H39B	109.7
C14—C13—H13B	108.7	H39A—C39—H39B	108.2
C12—C13—H13B	108.7	C39—C40—H40A	109.5
H13A—C13—H13B	107.6	C39—C40—H40B	109.5
C13—C14—H14A	109.5	H40A—C40—H40B	109.5
C13—C14—H14B	109.5	C39—C40—H40C	109.5
H14A—C14—H14B	109.5	H40A—C40—H40C	109.5
C13—C14—H14C	109.5	H40B—C40—H40C	109.5
			
N3—Zn1—S1—C1	84.91 (10)	C12—N2—C8—S3	4.3 (3)
S3—Zn1—S1—C1	−102.05 (8)	Zn1—S4—C8—N2	179.3 (2)
S2—Zn1—S1—C1	−2.83 (8)	Zn1—S4—C8—S3	−0.32 (11)
S4—Zn1—S1—C1	−174.81 (8)	Zn1—S3—C8—N2	−179.31 (18)
N3—Zn1—S2—C1	−122.35 (9)	Zn1—S3—C8—S4	0.35 (12)
S3—Zn1—S2—C1	121.38 (8)	C8—N2—C9—C10	−87.7 (3)
S1—Zn1—S2—C1	2.88 (8)	C12—N2—C9—C10	89.4 (2)
S4—Zn1—S2—C1	34.02 (12)	N2—C9—C10—C11	−173.2 (2)
N3—Zn1—S3—C8	90.13 (10)	C8—N2—C12—C13	−82.4 (3)
S1—Zn1—S3—C8	−83.78 (8)	C9—N2—C12—C13	100.6 (2)
S2—Zn1—S3—C8	−163.92 (7)	N2—C12—C13—C14	−53.5 (3)
S4—Zn1—S3—C8	−0.21 (7)	C19—N3—C15—C16	1.3 (3)
N3—Zn1—S4—C8	−111.38 (9)	Zn1—N3—C15—C16	179.25 (17)
S3—Zn1—S4—C8	0.22 (8)	N3—C15—C16—C17	−1.1 (4)
S1—Zn1—S4—C8	121.93 (8)	C15—C16—C17—C18	0.2 (3)
S2—Zn1—S4—C8	92.17 (11)	C15—C16—C17—C20	177.9 (2)
N4—Zn2—S5—C27	83.52 (10)	C16—C17—C18—C19	0.5 (4)
S7—Zn2—S5—C27	−91.36 (8)	C20—C17—C18—C19	−177.2 (2)
S8—Zn2—S5—C27	−168.71 (8)	C15—N3—C19—C18	−0.5 (4)
S6—Zn2—S5—C27	−0.27 (8)	Zn1—N3—C19—C18	−178.53 (19)
N4—Zn2—S6—C27	−123.49 (9)	C17—C18—C19—N3	−0.4 (4)
S5—Zn2—S6—C27	0.28 (8)	C16—C17—C20—C21	−94.6 (3)
S7—Zn2—S6—C27	122.90 (8)	C18—C17—C20—C21	83.0 (3)
S8—Zn2—S6—C27	63.46 (13)	C17—C20—C21—C22	−179.6 (2)
N4—Zn2—S7—C34	90.49 (10)	C20—C21—C22—C23	−122.9 (2)
S5—Zn2—S7—C34	−94.13 (8)	C20—C21—C22—C26	57.3 (3)
S8—Zn2—S7—C34	−1.74 (7)	C26—C22—C23—C24	0.8 (3)
S6—Zn2—S7—C34	−170.32 (8)	C21—C22—C23—C24	−179.1 (2)
N4—Zn2—S8—C34	−109.09 (9)	C25—N4—C24—C23	−1.0 (3)
S5—Zn2—S8—C34	124.24 (8)	Zn2—N4—C24—C23	−179.50 (18)
S7—Zn2—S8—C34	1.78 (8)	C22—C23—C24—N4	0.2 (4)
S6—Zn2—S8—C34	63.91 (12)	C24—N4—C25—C26	0.7 (3)
S3—Zn1—N3—C19	90.38 (18)	Zn2—N4—C25—C26	179.25 (18)
S1—Zn1—N3—C19	−96.06 (18)	N4—C25—C26—C22	0.3 (4)
S2—Zn1—N3—C19	−20.87 (18)	C23—C22—C26—C25	−1.0 (3)
S4—Zn1—N3—C19	165.37 (17)	C21—C22—C26—C25	178.8 (2)
S3—Zn1—N3—C15	−87.53 (18)	C31—N5—C27—S6	174.39 (17)
S1—Zn1—N3—C15	86.03 (18)	C28—N5—C27—S6	−1.5 (3)
S2—Zn1—N3—C15	161.22 (17)	C31—N5—C27—S5	−6.4 (3)
S4—Zn1—N3—C15	−12.54 (18)	C28—N5—C27—S5	177.64 (16)
S5—Zn2—N4—C24	−41.6 (2)	Zn2—S6—C27—N5	178.8 (2)
S7—Zn2—N4—C24	133.80 (17)	Zn2—S6—C27—S5	−0.41 (11)
S8—Zn2—N4—C24	−150.88 (18)	Zn2—S5—C27—N5	−178.76 (18)
S6—Zn2—N4—C24	30.66 (18)	Zn2—S5—C27—S6	0.45 (13)
S5—Zn2—N4—C25	139.88 (16)	C27—N5—C28—C29	−89.1 (3)
S7—Zn2—N4—C25	−44.70 (19)	C31—N5—C28—C29	94.7 (2)
S8—Zn2—N4—C25	30.62 (18)	N5—C28—C29—C30	179.3 (2)
S6—Zn2—N4—C25	−147.83 (17)	C27—N5—C31—C32	−89.0 (3)
C5—N1—C1—S2	2.1 (3)	C28—N5—C31—C32	87.1 (2)
C2—N1—C1—S2	178.98 (18)	N5—C31—C32—C33	174.1 (2)
C5—N1—C1—S1	−177.72 (17)	C38—N6—C34—S8	−179.81 (16)
C2—N1—C1—S1	−0.9 (3)	C35—N6—C34—S8	5.8 (3)
Zn1—S2—C1—N1	175.9 (2)	C38—N6—C34—S7	0.3 (3)
Zn1—S2—C1—S1	−4.24 (11)	C35—N6—C34—S7	−174.08 (17)
Zn1—S1—C1—N1	−175.59 (19)	Zn2—S8—C34—N6	177.6 (2)
Zn1—S1—C1—S2	4.54 (12)	Zn2—S8—C34—S7	−2.57 (11)
C1—N1—C2—C3	103.4 (3)	Zn2—S7—C34—N6	−177.32 (18)
C5—N1—C2—C3	−79.6 (3)	Zn2—S7—C34—S8	2.82 (12)
N1—C2—C3—C4	−65.2 (3)	C34—N6—C35—C36	−112.6 (2)
C1—N1—C5—C6	87.4 (3)	C38—N6—C35—C36	72.7 (3)
C2—N1—C5—C6	−89.7 (3)	N6—C35—C36—C37	63.7 (3)
N1—C5—C6—C7	176.1 (2)	C34—N6—C38—C39	−84.9 (3)
C9—N2—C8—S4	1.6 (3)	C35—N6—C38—C39	90.0 (2)
C12—N2—C8—S4	−175.34 (16)	N6—C38—C39—C40	171.8 (2)
C9—N2—C8—S3	−178.76 (16)		

### Hydrogen-bond geometry (Å, °)

**Table:**

*D*—H···*A*	*D*—H	H···*A*	*D*···*A*	*D*—H···*A*
C28—H28a···S1^i^	0.99	2.82	3.706 (3)	149
C40—H40b···S3^ii^	0.98	2.87	3.834 (3)	170

Symmetry codes: (i) *x*, −*y*+3/2, *z*−1/2; (ii) −*x*+1, *y*+1/2, −*z*+1/2.
